# Identifying individuals with rare disease variants by inferring shared ancestral haplotypes from SNP array data

**DOI:** 10.1093/nargab/lqaf033

**Published:** 2025-04-04

**Authors:** Erandee Robertson, Bronwyn E Grinton, Karen L Oliver, Liam G Fearnley, Michael S Hildebrand, Lynette G Sadleir, Ingrid E Scheffer, Samuel F Berkovic, Mark F Bennett, Melanie Bahlo

**Affiliations:** Population Health and Immunity Division, The Walter and Eliza Hall Institute of Medical Research, Parkville, Victoria 3052, Australia; Department of Medical Biology, University of Melbourne, Parkville, Victoria 3052, Australia; Population Health and Immunity Division, The Walter and Eliza Hall Institute of Medical Research, Parkville, Victoria 3052, Australia; Department of Medical Biology, University of Melbourne, Parkville, Victoria 3052, Australia; Epilepsy Research Centre, Department of Medicine, Austin Health, University of Melbourne, Heidelberg, Victoria 3084, Australia; Population Health and Immunity Division, The Walter and Eliza Hall Institute of Medical Research, Parkville, Victoria 3052, Australia; Department of Medical Biology, University of Melbourne, Parkville, Victoria 3052, Australia; Epilepsy Research Centre, Department of Medicine, Austin Health, University of Melbourne, Heidelberg, Victoria 3084, Australia; Population Health and Immunity Division, The Walter and Eliza Hall Institute of Medical Research, Parkville, Victoria 3052, Australia; Department of Medical Biology, University of Melbourne, Parkville, Victoria 3052, Australia; Epilepsy Research Centre, Department of Medicine, Austin Health, University of Melbourne, Heidelberg, Victoria 3084, Australia; Murdoch Children’s Research Institute, Royal Children’s Hospital, Parkville, Victoria 3052, Australia; Department of Paediatrics and Child Health, University of Otago, Wellington South 6242, New Zealand; Epilepsy Research Centre, Department of Medicine, Austin Health, University of Melbourne, Heidelberg, Victoria 3084, Australia; Murdoch Children’s Research Institute, Royal Children’s Hospital, Parkville, Victoria 3052, Australia; Department of Paediatrics, The University of Melbourne, Royal Children’s Hospital, Parkville, Victoria 3052, Australia; Florey Institute of Neuroscience and Mental Health, Heidelberg, Victoria 3084, Australia; Epilepsy Research Centre, Department of Medicine, Austin Health, University of Melbourne, Heidelberg, Victoria 3084, Australia; Population Health and Immunity Division, The Walter and Eliza Hall Institute of Medical Research, Parkville, Victoria 3052, Australia; Department of Medical Biology, University of Melbourne, Parkville, Victoria 3052, Australia; Epilepsy Research Centre, Department of Medicine, Austin Health, University of Melbourne, Heidelberg, Victoria 3084, Australia; Population Health and Immunity Division, The Walter and Eliza Hall Institute of Medical Research, Parkville, Victoria 3052, Australia; Department of Medical Biology, University of Melbourne, Parkville, Victoria 3052, Australia

## Abstract

We describe FoundHaplo, an identity-by-descent algorithm that can be used to screen untyped disease-causing variants using single nucleotide polymorphism (SNP) array data. FoundHaplo leverages knowledge of shared disease haplotypes for inherited variants to identify those who share the disease haplotype and are, therefore, likely to carry the rare [minor allele frequency (MAF) ≤ 0.01%] variant. We performed a simulation study to evaluate the performance of FoundHaplo across 33 disease-harbouring loci. FoundHaplo was used to infer the presence of two rare (MAF ≤ 0.01%) pathogenic variants, *SCN1B* c.363C>G (p.Cys121Trp) and *WWOX* c.49G>A (p.E17K), which can cause mild dominant and severe recessive epilepsy, respectively, in the Epi25 cohort and the UK Biobank. FoundHaplo demonstrated substantially better sensitivity at inferring the presence of these rare variants than existing genome-wide imputation. FoundHaplo is a valuable screening tool for searching disease-causing variants with known founder effects using only SNP genotyping data. It is also applicable to nonhuman applications and nondisease-causing traits, including rare-variant drivers of quantitative traits. The FoundHaplo algorithm is available at https://github.com/bahlolab/FoundHaplo (DOI:10.5281/zenodo.8058286).

## Graphical Abstract

**Graphical Abstract:**
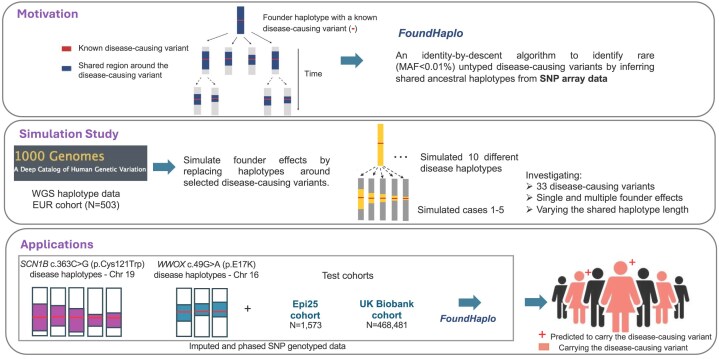

## Introduction

Detecting disease-causing variants (DCVs) is essential for identifying individuals at high risk for disease [1, 2], enabling appropriate patient care. Shared pathogenic variants observed in unrelated individuals or families may result from the variant arising independently or may be the result of a variant being inherited from a common ancestor. This leads to shared core haplotypes among carriers, a phenomenon known as a founder effect [3–10]. DCVs with founder events thus have an associated disease haplotype inherited from the common ancestor, shared by all the descendant variant carriers in subsequent generations [3–10]. Pathogenic variants initially thought to be recurrent have been able to be reclassified as inherited based on haplotype sharing from a common ancestral founder [6, 10]. Founder effects are also of great interest in any recombining genome for identifying bottlenecks, genetic diversity, selection, and breeding potential [11–13]. Shared haplotypes inherited from a common ancestor are defined as being identical by descent (IBD). Haplotypes shared by carriers of the DCV decrease in size over generations due to recombination events [3–10]. Regardless of the time elapsed since the original founder event, an IBD segment persists in current-day descendants carrying the DCV. This suggests that detecting associated disease haplotypes through an IBD approach can also infer the presence of these inherited DCVs.

Founder events for many DCVs have been previously described. Supplementary Table S1 lists an illustrative set of inherited genetic disorders with reported founder effects. Some genetic disorders show evidence of multiple founder events, each with its own unique haplotype. Examples of founder events include the Huntington’s disease repeat expansion (OMIM: 143100), which displays multiple founder events [7, 14–16], the *CFTR* p.F508del Cystic fibrosis variant (OMIM: 219700) [17], and the *GOSR2* p.G144W progressive myoclonus epilepsy-causing variant (OMIM: 604027) [18]. In general, the population frequency of these highly or fully penetrant DCVs is low, with a minor allele frequency (MAF) < 1%, typically leading to rare diseases. This low MAF typically leads to these variants being excluded in genome-wide association studies (GWAS).

Most published IBD methods seek to identify genome-wide IBD tracts rather than directly screening individuals for DCVs [19–25]. They do not make use of DCV haplotype information. Imputation platforms such as the Michigan Imputation Server (MIS) [26] and the TOPMed server [27] utilize linkage disequilibrium (LD) for genome-wide imputation of millions of variants not directly genotyped by single nucleotide polymorphism (SNP) arrays. However, these rely on having DCV haplotypes in their reference databases to impute DCVs, which is often not the case. Imputation performance also decreases rapidly with lower MAF < 0.01% [27, 28]. This highlights the necessity of exploring alternative approaches that are specifically designed to identify rare DCVs that enable or enhance the detection and analysis of rare variants with MAF ≤ 0.01%.

Here, we introduce FoundHaplo, an IBD-based tool developed using a first-order hidden Markov model (HMM) to screen rare DCVs with known founder effects from shared disease haplotypes requiring only SNP genotyping array data. FoundHaplo leverages its inference on pre-existing information of the location and haplotype of the DCV. This tool is particularly relevant given the widespread use of SNP genotyping arrays in Illumina technology for many species, GWAS to patient cohorts and large biobanks such as the UK Biobank (UKBB) [29], where many individuals are genotyped but not sequenced due to the relatively high costs of genome sequencing in contrast to SNP genotyping arrays or due to a lack of remaining biospecimens suitable for sequencing [30, 31]. Even though SNP genotyping arrays are cost-effective and commonly available, many DCVs are not captured directly on SNP arrays. FoundHaplo addresses the gap in screening DCVs not directly SNP genotyped or imputable with existing tools due to their low MAF and lack of representation in large databases leveraged for imputation [32–35].

We perform a comprehensive simulation study to demonstrate the performance of FoundHaplo under single and multiple founder effects and then apply the algorithm to identify DCVs in cohorts, including the UKBB [29], demonstrating that FoundHaplo is a useful screening tool, which could be applied to bespoke catalogues of DCV haplotypes to identify individuals that merit further sequencing.

## Materials and methods

### FoundHaplo HMM

**Figure 1.**
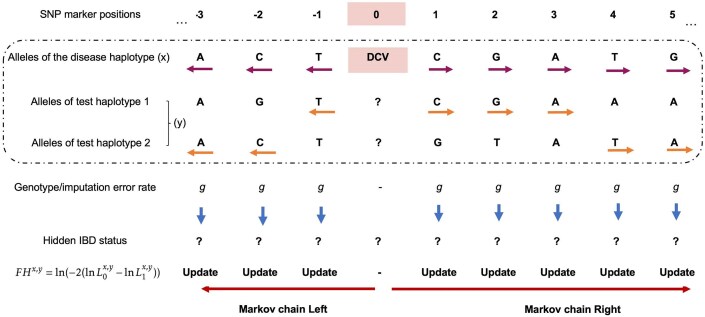
Calculating the *FH* score for a disease-test pair based on the HMM approach. IBD status is unknown at all the SNP markers, making IBD segment boundaries unknown. We test a disease haplotype against an individual’s two haplotypes, observing alleles surrounding the DCV and moving from the DCV to the left and right. The algorithm updates the likelihood of hidden IBD (L_0_ for the null and L_1_ for the alternate hypothesis) in the *FH* score. It accounts for a fixed rate of genotype and imputation errors and switches between the test individual’s two haplotypes to handle phasing errors.

The FoundHaplo HMM aims to differentiate between random haplotype sharing and IBD between a known disease haplotype and a test individual in the vicinity of a DCV in a hypothesis-testing framework to infer the presence of a DCV in an individual’s phased and imputed genotyping data. The null hypothesis (H0) asserts no IBD between the individual’s haplotypes and the disease haplotype, indicating no common founder inheritance of the DCV. The alternative hypothesis (H1) suggests at least one haplotype presents evidence of IBD with the disease haplotype, indicating inheritance from a common founder. The FoundHaplo HMM, focusing on biallelic SNPs, models IBD to determine the hidden IBD state, discerning between no IBD (0) and IBD (1) based on the observed reference or alternate (0 or 1) alleles. HMMs in FoundHaplo replace the typical “waiting time” with the genetic map distance in Morgans from a known DCV locus to the next recombination event. With unknown IBD sharing boundaries, the algorithm starts Markov chains at the DCV locus and extends in opposite directions, comprising two Markov chains as illustrated in Fig. 1.

The FoundHaplo algorithm calculates the log-likelihood ratio (LLR) of IBD versus non-IBD at each genetic marker surrounding the DCV (denoted by marker 0) for a disease haplotype and a test pair of imputed haplotypes. The likelihood of IBD is encapsulated in the FoundHaplo (*FH)* score, defined as ln(LLR) (Supplementary Note S1).

Genotype and imputation errors are indistinguishable in this model and are treated similarly, assumed to occur at a fixed, uniform rate *g* (1% by default) across the genome [36, 37] (Fig. 1, Supplementary Fig. S1, Supplementary Note S1, and Supplementary Table S2). Genotype markers with missing values are excluded from the analysis. The algorithm does not incorporate LD since IBD segments are typically larger than the length of LD blocks.

To propagate the LLR, the FoundHaplo algorithm switches to the alternate haplotype of the test individual when haplotype sharing ceases on the current one, as depicted by horizontal arrows in the dashed box in Fig. 1. This approach captures potential sharing on the other haplotype and accommodates block phasing errors introduced in the test haplotypes by LD-based phasing tools. We used a comprehensive simulation study to evaluate the FoundHaplo algorithm and the criteria to terminate the Markov chains (Supplementary Fig. S2). The Markov chains end when sharing around the DCV between the disease haplotype and test individual stops (Supplementary Fig. S3 and Supplementary Note S1). When multiple known disease haplotypes for a single disease exist, the *FH* score is determined as the maximum of individual *FH* scores across all available disease haplotypes for that variant (Supplementary Figs S4 and S5 and Supplementary Note S2).

FoundHaplo can only identify evidence of DCVs if the haplotypes on which the DCVs are located are represented in the set of disease haplotypes available for testing. Hence, FoundHaplo is a screening test for DCVs. It cannot exhaustively identify DCVs.

### Using empirical *P*-values for *FH* score evaluation

A fraction of controls could give medium to high *FH* scores even though they do not share with disease haplotypes (Supplementary Figs S6 and S7). Therefore, in the FoundHaplo algorithm, while the LLR test statistics under the null hypothesis are theoretically asymptotically chi-square distributed [38], the actual distribution deviates due to LD (Supplementary Fig. S8). Therefore, the significance of the FoundHaplo statistic is assessed using the empirical distribution of *FH* scores from a control population. A test individual is identified as having IBD sharing with a disease haplotype if their *FH* score exceeds a critical threshold, typically set based on the 99th percentile of the *FH* score distribution in a control cohort of the same ancestral population as the test cohort using data from the 1000 Genomes Phase 3 haplotypes [39].

### Inputs required by FoundHaplo

The algorithm tests each accumulated disease haplotype against the two haplotypes of all the test individuals. FoundHaplo outputs the *FH* score for each disease-test pair for each of the DCVs examined. An imputation step is used to increase marker density prior to applying FoundHaplo. Imputation and phasing can be performed jointly using LD-based genome-wide imputation and phasing tools, or with servers such as the MIS [26] or TOPMed server [27].

FoundHaplo requires phased disease haplotypes, best achieved through pedigree phasing with another confirmed carrier of the same DCV from the same family. This avoids errors from LD-based genome-wide phasing, which only resolves phasing to LD block resolution. Additionally, individuals with long homozygosity regions due to related parents can be used as the source of recessive disease haplotypes, as the homozygosity tracts are often much longer than any shared IBD tracts.

FoundHaplo algorithm relies on two parameters: minor allele frequencies (MAF) of genetic markers and ancestry and sex agnostic recombination rates between markers. Ancestry and sex agnostic recombination rates for the human genome are commonly used in IBD algorithms [5, 9, 25].

The FoundHaplo algorithm, available as an R package with a disease haplotype database schema, is freely available at https://github.com/bahlolab/FoundHaplo. Researchers can create and manage their own database instances of disease haplotypes, maintaining data confidentiality while running FoundHaplo. A detailed mathematical derivation of the algorithm is provided in the Supplementary Note S1.

### Simulation study

We performed a simulation study for 33 DCVs to evaluate the performance of the FoundHaplo algorithm using 503 unrelated individuals with European (EUR) ancestry from the 1000 Genomes Project Phase 3 dataset [39]. Most of the DCVs we simulated are located at known repeat expansion (RE) loci (Supplementary Tables S3 and S4). REs are rare, often inherited with known founder effects [3, 4, 8, 40]. REs are caused by short tandem repeat (STR) expansions, and they exhibit high heterozygosity rates due to their rapid mutation rates [41]. Even though SNPs tag STRs with high LD, multiallelic repeats are often not in LD with common SNPs [42]. Therefore, the direct relationship between STRs and SNP haplotypes can be complex since LD patterns at STRs vary widely due to factors such as repeat length and mutation rate, making REs harder to detect using SNP genotyping array data [43, 44].

REs are, therefore, an excellent candidate set of diseases to demonstrate the utility of FoundHaplo. The simulation study investigated (i) single founder effects, where multiple different versions of a single disease haplotype are found in the present time that are all distantly related and descended from a single ancestor, and (ii) multiple founder effects, where the same DCV has arisen independently in multiple unrelated founders, resulting in multiple unique haplotypes (Supplementary Fig. S2). Test cohorts were constructed by designating a fraction of individuals as cases. Each case was simulated by replacing the haplotype spanning the DCV locus with a randomly selected disease haplotype to simulate the presence of an inherited DCV. Cases were simulated to share different sizes of the disease haplotypes (0.5, 1, 2, and 5 cM) surrounding the DCV to simulate pairs of individuals with varying times to the most recent common ancestor (Supplementary Fig. S2). The rest of the test cohort remained unchanged, acting as controls. In our simulations, we introduced genotype and imputation errors by altering 1% of marker alleles genome-wide. Additionally, we simulated phasing errors in all individuals (except those used to derive the disease haplotypes) by switching blocks of adjacent marker alleles to the alternate haplotype with a rate of one switch per 20.05 Mbp [45].

For each of the 33 simulated disease loci, we created 10 founder scenarios, generating 10 disease haplotypes and 50 cases (5 per disease haplotype) for each scenario. This resulted in 1320 simulation datasets encompassing both single and multiple founder effects with varying sharing lengths (Supplementary Table S3 and Supplementary Algorithm S3).

### Detecting the *SCN1B* c.363C>G and *WWOX* c.49G>A rare epilepsy variants

FoundHaplo was used to predict carriers of the *SCN1B* c.363C>G (p.Cys121Trp) (OMIM: 604233) and *WWOX* c.49G>A (p.E17K) (OMIM: 616211) rare variants in two cohorts. Based on the gnomAD version v4.1.0, *SCN1B* c.363C>G has a MAF of 0.01047% in gnomAD [46, 47] and causes autosomal dominant genetic epilepsy with febrile seizures plus [48–51]*. WWOX* c.49G>A has a MAF of 0.01037% in gnomAD [46, 47] and causes autosomal recessive developmental and epileptic encephalopathy [52–54].

Cohort 1 consisted of 1573 individuals with different types of epilepsy recruited in Australia or New Zealand as part of the international Epi25 study [55]. Cohort 2 is the UKBB cohort (*n* = 468 481) accessed through project ID 36610 [29]. Both cohorts, primarily of EUR ancestry and with whole exome sequencing (WES) and SNP genotyping data (Supplementary Note S3), identified two individuals in the Epi25 cohort and 171 individuals in the UKBB cohort who carried the *SCN1B* c.363C>G variant and 172 individuals in the UKBB who carried the *WWOX* c.49G>A variant.

For FoundHaplo analysis, five *SCN1B* c.363C>G and three *WWOX* c.49G>A disease haplotypes were created using duo and trio genotype data of eight different families (Supplementary Note S3). The Epi25, UKBB cohorts, and *SCN1B* c.363C>G and *WWOX* c.49G>A disease haplotypes were prepared from SNP genotyping data processed using standard quality control steps. Samples with a call rate of <98% and SNPs with an overall call rate of <98% were removed, and data were harmonized to 1000 Genomes data using Genotype Harmonizer [56]. Imputation was performed using the MIS [26] with the EUR cohort of the 1000 Genomes Phase 3 haplotypes (hg19 human genome build) [39] as the reference panel. As the UKBB cohort was already imputed, samples only needed to be phased to run FoundHaplo, which was performed in-house using SHAPEIT4 (version 4.2.2) [57]. MAFs were annotated with gnomAD population frequencies [46]. The dataset was trimmed to contain a 20-cM region around the DCVs with 10 cM on either side of the respective DCV loci. Only biallelic markers with an imputation quality score ≥ 0.3 were retained. The *SCN1B* c.363C>G and *WWOX* c.49G>A families were pedigree phased to extract five disease haplotypes for *SCN1B* c.363C>G and three disease haplotypes for *WWOX* c.49G>A variant to be tested on the Epi25 cohort and the UKBB cohort.

The EUR cohort of 1000 Genomes Phase 3 [39] was used as the control cohort when using FoundHaplo. None of the samples in the EUR cohort of the 1000 Genomes data carried either of the two variants.

FoundHaplo predictions were computed using the 99th percentile critical value from the 1000 Genomes data. The algorithm’s effectiveness was evaluated using the area under the precision-recall (PR) curve (AUPRC), appropriate for imbalanced datasets [58, 59]. The performance of a random classifier of a PR curve can be evaluated with the baseline rate, which is the ratio of positives to the total cohort size [58, 59].

This study was approved by the Austin Health Human Research Ethics Committee. Informed consent was obtained and archived from all participants or their legal guardian. Research was approved by the Human Research Ethics Committee at The Walter and Eliza Hall Institute of Medical Research (G20/01, 17/09LR).

### Web resources

BCFtools, https://samtools.github.io/bcftools/bcftools.html

FoundHaplo, https://github.com/bahlolab/FoundHaplo

Genotype Harmonizer, https://github.com/molgenis/systemsgenetics/wiki/Genotype-Harmonizer

gnomAD, https://gnomad.broadinstitute.org/

HapMap project, https://www.genome.gov/10001688/international-hapmap-project

Michigan Imputation Server, https://imputationserver.sph.umich.edu/

OMIM, http://www.omim.org/

Plink 1.9, https://www.cog-genomics.org/plink/1.9/

SHAPEIT4, https://odelaneau.github.io/shapeit4/

TOPMed Imputation Server, https://imputation.biodatacatalyst.nhlbi.nih.gov/

UK Biobank, https://www.ukbiobank.ac.uk/

VCFtools, https://vcftools.github.io/index.html

1000 Genomes Project, https://www.internationalgenome.org/

## Results

### Simulation study

We performed a simulation study for 33 DCVs using the EUR cohort of the 1000 Genomes Phase 3 data to test FoundHaplo’s ability to identify shared disease haplotypes. The simulation study investigated single and multiple founder effects for haplotype sharing of 0.5, 1, 2, and 5 cM (Supplementary Fig. S2). The predictions were made by evaluating the *FH* score of each disease-test pair against the *FH* score distribution of the control cohort at the 99th critical value percentile.

**Figure 2.**
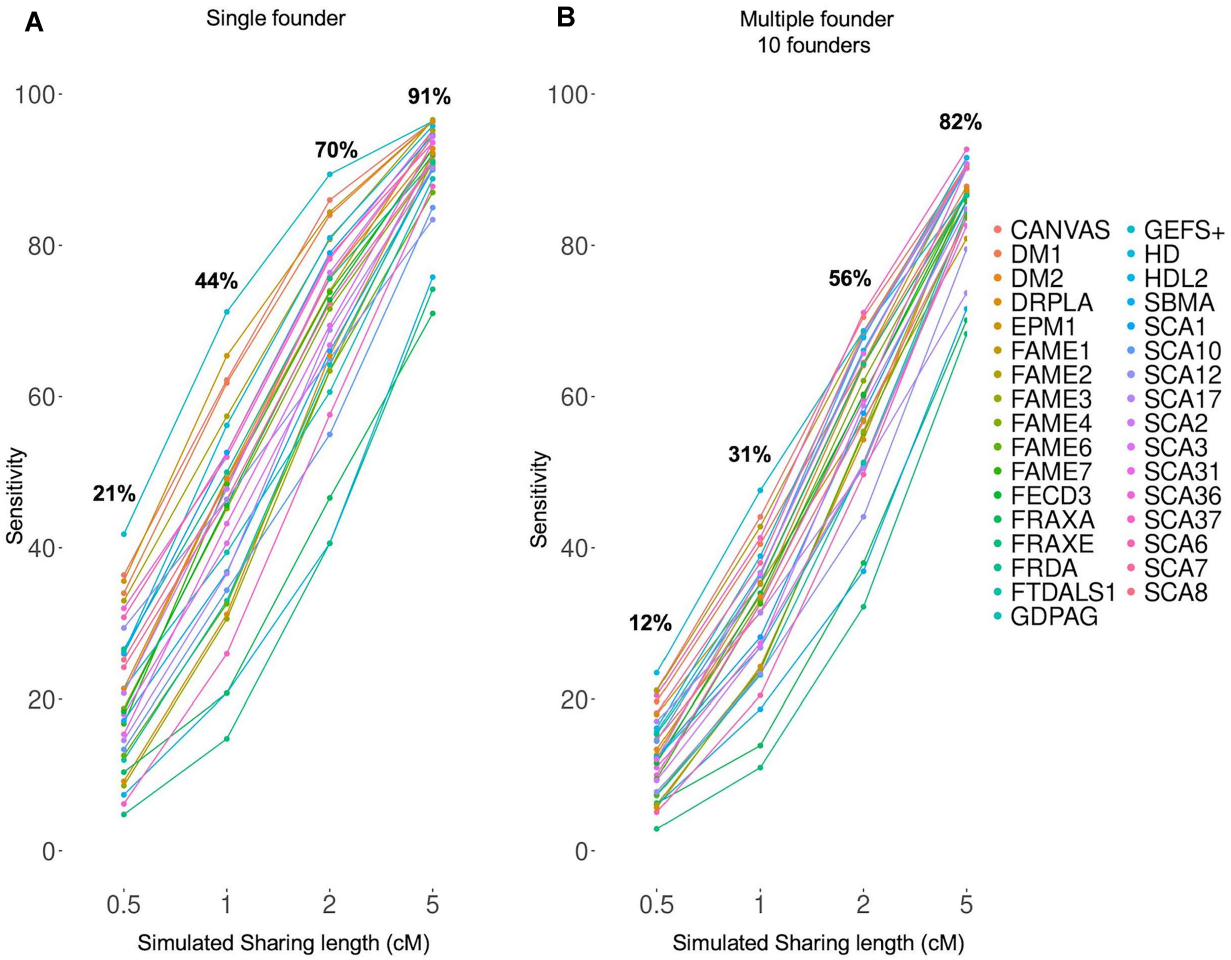
Sensitivity of the FoundHaplo algorithm for 33 simulated disease loci at the default 99th percentile critical value. The sensitivity for (**A**) single founder effects and (**B**) multiple founder effects were calculated based on the ability to correctly predict simulated cases that have a common ancestor with the 10 disease haplotypes in use.

Fig. 2 displays FoundHaplo’s sensitivity at the default 99th percentile, measured by its ability to identify simulated cases sharing an ancestor with the disease haplotypes in use. The algorithm showed 81% sensitivity for single founder effects and 69% for multiple founder effects with a shared length of at least 2 cM, maintaining an empirical false positive rate of 1%. Sensitivity is higher for single founder effects due to the ten disease haplotypes having a shared core ancestral haplotype. The sensitivity increases with larger simulated shared segments that can be better differentiated from the general population, indicative of a more recent common ancestor.

**Figure 3.**
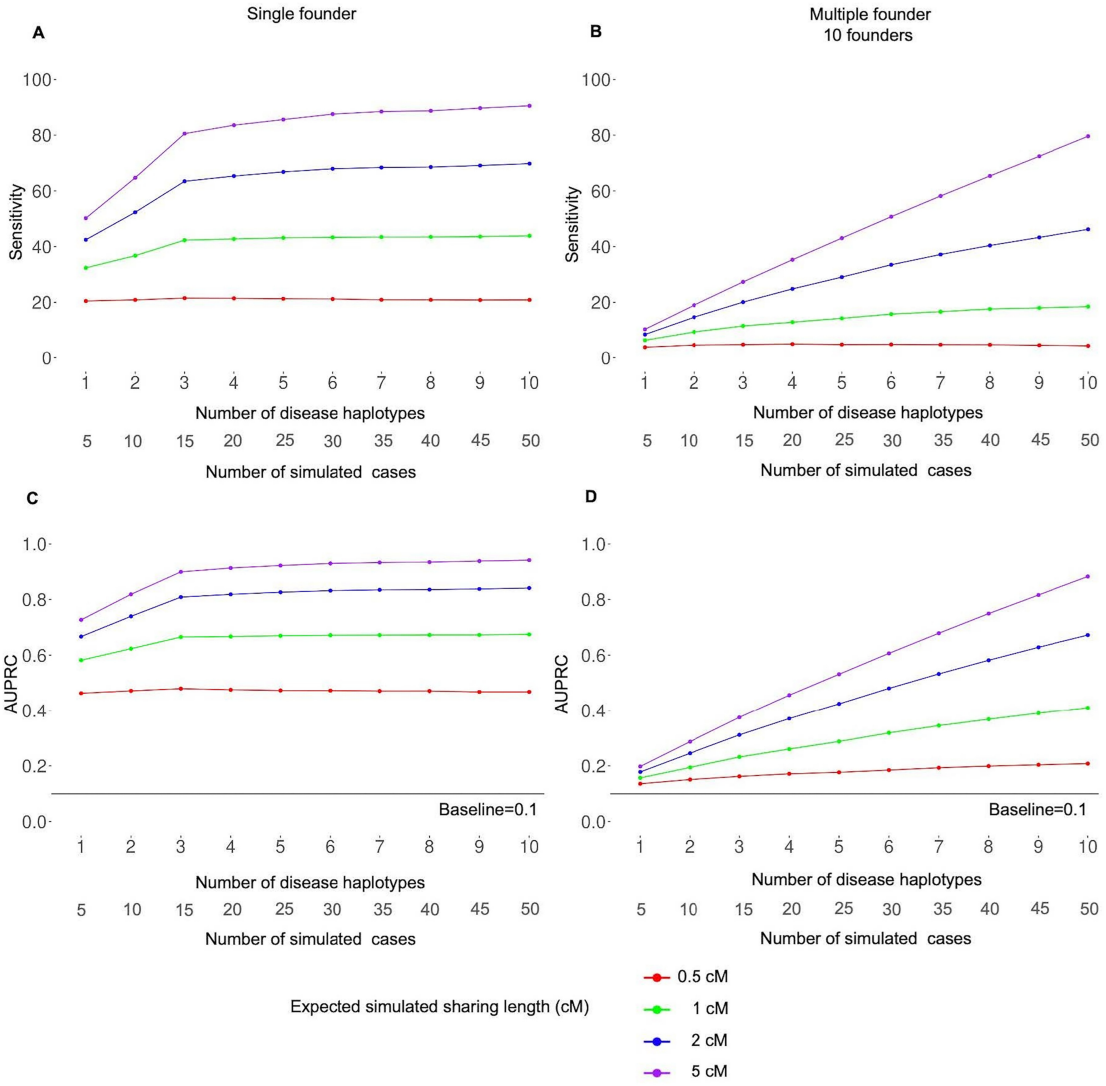
Performance of the FoundHaplo algorithm based on sensitivity and AUPRC by the number of disease haplotypes used averaged for all 33 simulated disease loci. The (**A**) sensitivity for single founder effects, (**B**) sensitivity for multiple founder effects, (**C**) AUPRC for single founder effects, and (**D**) AUPRC for multiple founder effects were calculated based on the ability to correctly predict all the simulated cases in simulated test cohorts. The AUPRC of a random classifier in each simulation is 0.1 and is shown in black horizontal lines in panels (C) and (D).

Fig. 3 demonstrates the variation in sensitivity and AUPRC with increasing numbers of disease haplotypes. Sensitivity and AUPRC were calculated based on accurately predicting all simulated cases in each test cohort. Five cases were simulated for each of the 10 disease haplotypes; therefore the AUPRC of a random classifier in each simulation is 0.1 (50 cases/492 total cohort size). The AUPRC for a random classifier is shown in black horizontal lines in Fig. 3C and D.

Sensitivity and AUPRC are greater for simulations based on single founder effects in FoundHaplo. The algorithm can not identify sharing between haplotypes without a common ancestor with known disease haplotypes, leading to lower sensitivity in multiple founder effects with fewer, distinct disease haplotypes. This illustrates how having more unique disease haplotypes enhances the performance, especially for DCVs with multiple founder effects, as shown in Fig. 3B and D.

The genotype and imputation error rate allowed in FoundHaplo is a parameter that can be set by the user (default = 1%). To quantify the robustness of FoundHaplo against misspecification of error rates, we extended the simulation study to test the performance of FoundHaplo when the assumed error rate incorporated in the model was higher or lower than the simulated error rate of 1%. We run FoundHaplo, setting the error rate parameter to 0.5%, 1%, and 2% using a randomly selected subset of the 33 DCV loci for single founder effects for the expected simulated sharing length of 5 cM at the 99th critical percentile.

Both mean and median AUPRC for FoundHaplo as shown by Supplementary Fig. S9, remain above 0.9 for all three 0.5%, 1%, and 2% values of genotype error rate parameter used in the algorithm, demonstrating that FoundHaplo is robust against misclassification of error rates.

FoundHaplo’s performance varies depending on the selected critical value percentile. A stricter critical percentile chosen in FoundHaplo decreases sensitivity (Supplementary Fig. S10). Performance further depends on the genomic location of the DCV loci and properties of the disease haplotypes. These include recombination rates in the DCV regions, with high background LD resulting in common haplotypes shared between individuals, diminishing the performance of FoundHaplo. The frequency of disease haplotypes also determines performance. The more unique the disease haplotypes, the lower the chances that they exist among the general population, allowing the algorithm to better identify individuals that share the DCV from a common founder. The 33 selected real DCV loci cover a broad spectrum of these features (described in Supplementary Table S4). Simulated DCV loci located on the X chromosome gave the lowest overall AUPRC due to a lower number of recombination events on the X chromosome (Supplementary Table S4).

Mean run time of the FoundHaplo HMM per locus for a disease-case pair for simulations with expected sharing lengths of 0.5, 1, 2, and 5 cM are 20, 24, 29, and 40 s, respectively. Since the total run time can be drastically reduced with parallelisation, FoundHaplo makes use of a scalable and reproducible scientific Nextflow pipeline to manage running pairs of disease-test or disease-control comparisons in parallel.

### Detecting individuals with the *SCN1B* c.363C>G and *WWOX* c.49G>A rare epilepsy variants

FoundHaplo was used to predict carriers of the *SCN1B* c.363C>G (p.Cys121Trp) (OMIM: 604233) and *WWOX* c.49G>A (p.E17K) (OMIM: 616211) rare variants in the Epi25 and the UKBB cohort.

The original five *SCN1B* c.363C>G (p.Cys121Trp) carriers shared a core haplotype of 4.1 cM around the *SCN1B* c.363C>G variant, and the three *WWOX* c.49G>A (p.E17K) carriers shared a core haplotype of 3.9 cM around the *WWOX* c.49G>A variant, suggesting a common ancestor between the families for each of the two variants (Supplementary Figs S11 and S12).

We compared the created disease haplotypes for these variants with the confirmed carriers based on WES analysis in the UKBB and Epi25 cohorts. All of the 178 *SCN1B* c.363C>G carriers shared a core haplotype of 55 kb. Among carriers in the Epi25 and the UKBB, a minimum pairwise sharing of 63 kb, a median of 4650 kb and a maximum of 12 321 kb was observed. All of the 175 *WWOX* c.49G>A carriers shared a core haplotype of 157 kb. Among carriers in the UKBB, a minimum pairwise sharing of 540 kb, a median of 1762 kb and a maximum of 10 000 kb was observed (Supplementary Figs S11 and S12). This suggests that all the carriers have a common ancestor for each of the two variants. The two Epi25 carriers shared the shortest genomic region with the core haplotype (63 kb and 373 kb) around the *SCN1B* c.363C>G locus, implying that they are more distantly related compared with the rest of the carriers.

**Figure 4.**
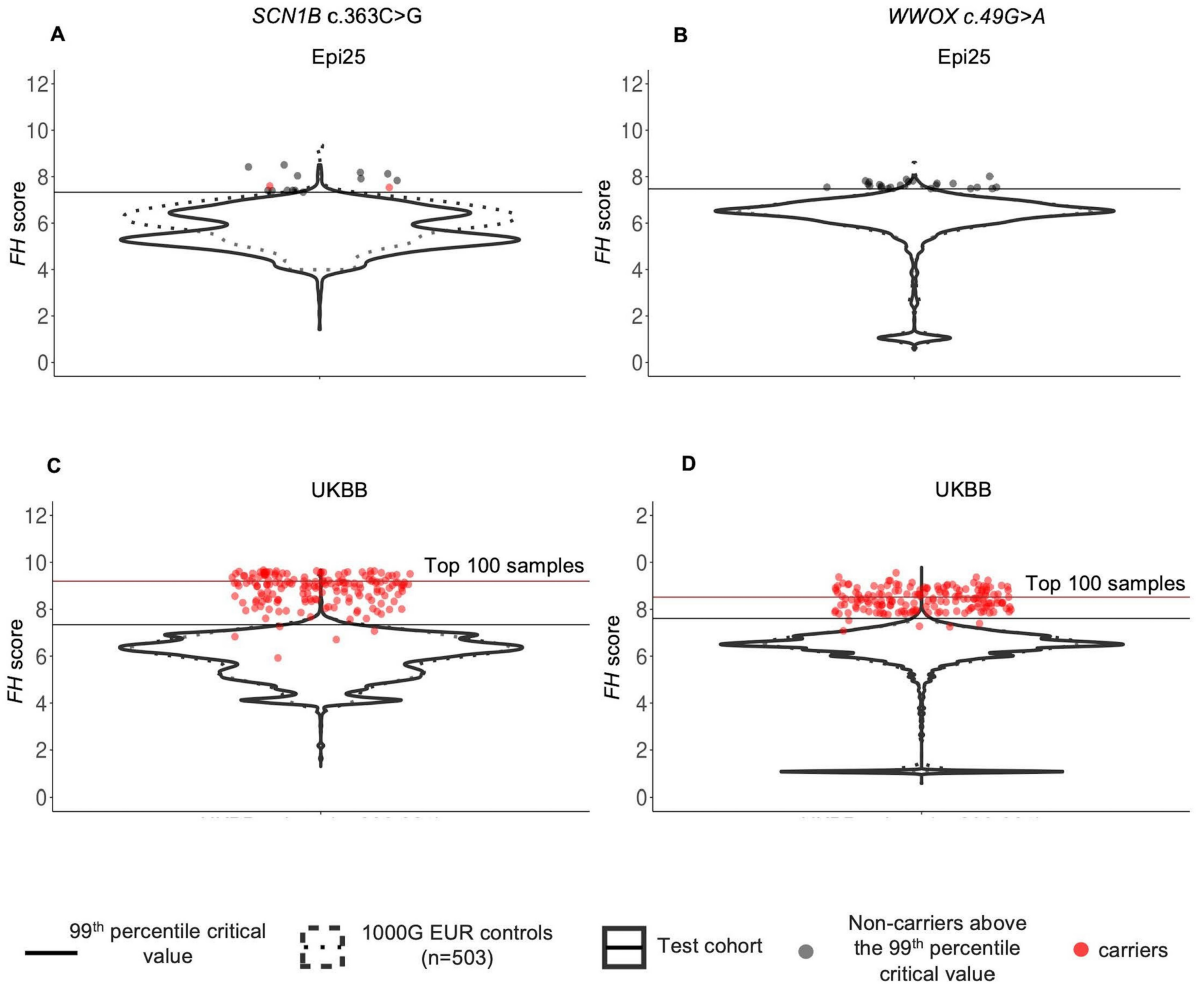
Distribution of *FH* scores in the Epi25 cohort and the UKBB cohort. Two disease variants are shown: (**A**) *SCN1B* c.363C>G variant in the Epi25 cohort, (**B**) *WWOX* c.49G>A variant in the Epi25 cohort, (**C**) *SCN1B* c.363C>G variant in the UKBB cohort, and (**D**) *WWOX* c.49G>A variant in the UKBB cohort. The distribution of *FH* scores in the test cohorts is shown with solid violin plots, and 1000 Genomes controls with dashed violin plots. The 1000 Genomes critical values at the 99th percentile are shown in horizontal lines. Samples confirmed to carry the variant based on WES analysis are shown in red. Samples without the variant that passed the 99th percentile critical value for the Epi25 cohort are represented in black. The remaining samples are not represented individually. The critical values corresponding to the top 100 samples for the UKBB cohort are shown in brown horizontal lines for the UKBB cohort.

The distribution of the *FH* scores in the Epi25 (*n* = 1573) and the UKBB cohorts (*n* = 468 481) and in their respective control cohorts for the constructed *SCN1B* c.363C>G and *WWOX* c.49G>A disease haplotypes are shown in Fig. 4. Noncarriers above the 99th percentile critical value are shown in black for the Epi25 but not for the UKBB cohort, due to the large number of individuals (*n* = 4685) at this percentile under the null hypothesis alone. Using the 99th percentile, FoundHaplo predicted both *SCN1B* c.363C>G variant carriers and 13 noncarriers (100% sensitivity and 0.8% false positive rate) in the Epi25 cohort. There are no *WWOX* c.49G>A carriers in the Epi25 cohort, but FoundHaplo identified 23 noncarriers (1.5% false positive rate). FoundHaplo predicted 166 *SCN1B* c.363C>G variant carriers (97% sensitivity and 2% false positive rate) and 167 *WWOX* c.49G>A carriers correctly (97% sensitivity and 0.9% false positive rate) in the UKBB cohort.

PR curve analysis was not done for the Epi25 cohort due to the limited number of *SCN1B* carriers and the absence of *WWOX* carriers. In the UKBB cohort, the AUPRC of a random classifier is 0.00 037 (carriers/total cohort size) for both variants [58, 59], whereas FoundHaplo achieved an AUPRC of 0.46 for the *SCN1B* c.363C>G variant and 0.6 for the *WWOX* c.49G>A variant, indicating its effectiveness in distinguishing carriers from noncarriers in the UKBB cohort.

The total number of predictions above the 99th percentile for the UKBB (*n* = 9523 for *SCN1B* c.363C>G and *n* = 4459 for *WWOX* c.49G>A) is typically too high for further screening (Supplementary Tables S5 and S6). For large cohorts, FoundHaplo can prioritise predictions by setting the selection of a specific number of samples with the highest *FH* scores for screening. Using this approach for the UKBB cohort, we assessed the top 100 samples for each of the two variants. This resulted in correctly predicting 53 carriers for the *SCN1B* c.363C>G variant and 74 carriers for the *WWOX* c.49G>A variant, with 31% and 43% sensitivity and with 53% and 74% true discovery rate, respectively (Supplementary Tables S5 and S6).

FoundHaplo required ∼30 min to complete the analysis of the *SCN1B* c.363C>G and *WWOX* c.49G>A variants in the Epi25 cohort and around 4 days to complete the analysis of the *SCN1B* c.363C>G and *WWOX* c.49G>A variants in the UKBB cohort. The UKBB cohort was run by splitting the cohort into subsets with a 1000 samples each, and each subset was run for the accumulated disease haplotypes using the Nextflow pipeline.

## Discussion

Achieving a genetic diagnosis is critical, providing opportunities for improved patient care by tailoring therapy appropriately and potentially impacting the diagnosis of other family members, including distant relatives, who may also be at risk [1, 2]. With declining costs and widespread use of SNP genotyping compared with whole genome sequencing or WES, there are SNP arrays for millions of individuals in public databases. Hidden in these data are likely many individuals who have inherited known, rare DCVs that are not ascertained directly by the SNP array and cannot be imputed due to their rarity. Thus, FoundHaplo enables the screening of known rare DCVs, increasing the utility of SNP genotyping data, and serves as a preliminary screening approach that can increase the likelihood of identifying individuals who may benefit from further genetic testing. FoundHaplo, uses SNP genotyping array data to identify individuals with rare DCVs (MAF ≤ 0.01%) based on known disease haplotypes, unlike traditional IBD algorithms that target genome-wide IBD regions [19–25]. In our simulation study, FoundHaplo successfully detected 75% of cases sharing at least 2 cM of a disease haplotype.

We evaluated the ability of FoundHaplo to identify two rare variants, *SCN1B* c.363C>G (p.Cys121Trp) and *WWOX* c.49G>A (p.E17K), that can cause epilepsy in two cohorts, Epi25 and the UKBB cohort. *SCN1B* c.363C>G (p.Cys121Trp) is a dominant allele, and *WWOX* c.49G>A (p.E17K) is a recessive variant requiring the presence of a second allele to cause disease. The two variants were neither genotyped on SNP arrays, nor imputed by the MIS [26] or the TOPMed server [27] in the Epi25 cohort, possibly due to the absence or scarcity of variant-carrying haplotypes in reference panels.

In the publicly available UKBB cohort, the *SCN1B* c.363C>G variant was imputed as heterozygous carriers in only nine samples out of 171 carriers (5% sensitivity) using the impute2 tool [60] with the HRC [61], UK10K [62], and 1000 Genomes Phase 3 [39] reference panels. The imputation quality based on the R-squared value of the *SCN1B* c.363C>G variant in the UKBB cohort was 0.45. The *WWOX* c.49G>A variant is not imputed in the publicly available UKBB cohort, likely due to there being no *WWOX* c.49G>A carriers in the reference panels used in imputation. In contrast, FoundHaplo was able to correctly predict 55 *SCN1B* c.363C>G carriers and 74 *WWOX* c.49G>A carriers, using the 99th critical percentile for the Epi25 and the top 100 samples for the UKBB cohort, showing a notable 37% sensitivity for both variants, which is a substantial improvement compared with 5% sensitivity in genome-wide imputation tools. It is likely that rare variant disease haplotypes with MAF ≤ 0.01% will never be represented in imputation cohorts at a sufficient number to facilitate imputation with reasonable sensitivity. However, genome-wide imputation tools achieve high accuracy for more common disease-causing variants.

We have shown that FoundHaplo can successfully identify carriers of rare variants using surrogate disease haplotypes; however, the algorithm has a number of limitations. It uses a fixed error rate for genotype and imputation (1% by default), regardless of MAF variations. A more refined method would adjust the error rate based on MAF, accommodating a higher error rate for rarer variants [34, 35, 63, 64].

FoundHaplo does not account for LD. While LD blocks are typically short and the algorithm targets longer IBD segments for the founder effects we seek to identify, incorporating LD might improve the detection of shorter IBD segments (≤1 cM) with older common ancestors. The effect of LD can be seen in the simulation results, with performance varying by disease locus due to differences in background haplotype sharing, caused by locally specific LD, between controls.

FoundHaplo can not identify carriers who have inherited the DCV from a different founder that is not represented by any of the accumulated disease haplotypes. The impact of this limitation can be minimized by accumulating unique disease haplotypes for disease variants identified with multiple founder effects, which results in increased accuracy and efficiency of the FoundHaplo algorithm based on the simulation study. We explored the effect of different numbers of founders in our simulation studies. Sensitivity and AUPRC are lower when there are multiple founders compared with just a single founder. As the number of founders increases, there will be more distinct disease haplotypes within the population, reducing the likelihood of capturing the full genetic diversity associated with the disease variant and decreasing the effectiveness of the algorithm.

FoundHaplo presumes accurate phasing of disease haplotypes, typically requiring multiple family members with a known DCV for pedigree phasing. Other LD-based genome-wide phasing approaches, like those in TopMed or MIS, only offer block phasing. Individuals with long homozygosity regions due to related parents can also be used as the source of recessive disease haplotypes, as the homozygosity tracts are often much longer than any shared IBD tracts. Additionally, FoundHaplo cannot determine the exact number of disease haplotype copies in a test individual. This is not relevant for autosomal dominant diseases since only one copy is sufficient to cause the disease. For recessive diseases, FoundHaplo can only predict individuals that carry at least one copy of the disease haplotype and further testing is required to determine the number of copies; however, this does not impact the utility of FoundHaplo as a screening tool. It will identify both carriers (one copy) and those individuals with two copies. These individuals may be homozygous or compound heterozygotes for inherited DCVs.

The power of the *FH* statistic increases the more unique disease haplotypes that are present. Additionally, FoundHaplo performs best when the disease and test individuals are more closely related to each other, allowing the preservation of a larger ancestral disease haplotype, and this is more likely to occur when there are more unique disease haplotypes present.

One important consideration when using FoundHaplo is the choice of critical threshold. The best choice depends on the appropriate balance between increasing sensitivity and minimizing the number of false positives. The “false positives” identified by FoundHaplo that do not share the DCV may still share the disease haplotype since FoundHaplo uses disease haplotypes as surrogates for DCVs. This depends on the time between the DCV mutation and the uniqueness of the DCV-carrying haplotype prior to the DCV arising and is always unknown. For example, the *SCN1B* c.363C>G variant-associated haplotype is present in ∼1% of the population [6], however, only a fraction of those individuals inherited the version of this haplotype with the DCV. Therefore, the FoundHaplo method is designed as a genomic screening tool that can flag individuals who may be carriers of pathogenic variants based on haplotype sharing. It does not aim to comprehensively identify all carriers in a population.

FoundHaplo is useful in screening for variants that are unable to be detected directly or after imputation from SNP microarray but is also applicable to short-read sequencing data for variants that are difficult to detect with short-read data. This includes complex structural variants and REs such as cerebellar ataxia with neuropathy and vestibular areflexia syndrome (CANVAS, OMIM: 614575) [4, 65]. FoundHaplo should be able to be used on any recombining genome for predicting inherited genetic variants. Identifying founder effects can provide insight into the strategies for endangered or invasive species, processes that shape biodiversity, the resilience of populations to environmental changes, genetic diversity of stocks and breeds, affecting traits like disease resistance and yield and origin, and the evolution of drug resistance for diseases like malaria [11–13, 66].

The novelty of the FoundHaplo approach lies in using prior knowledge of known disease haplotypes to find local IBD segments specific to disease-causing variants of interest. We demonstrated the ability of FoundHaplo to detect two inherited rare variants that cause epilepsy. There are many other similar founder effects ideally suited for screening with this method. FoundHaplo could significantly aid in identifying carriers of known disease variants using SNP array data who might otherwise be unlikely to undergo targeted genetic testing or receive a genetic diagnosis.

## Supplementary Material

lqaf033_Supplemental_File

## Data Availability

Data used in the simulations can be accessed from 1000 Genomes Phase 3, which is publicly available. This research has been conducted using data from UK Biobank, a major biomedical database. The UK Biobank is an open access resource. To access the UKBB datasets, users need to register as a UKBB researcher (https://www.ukbiobank.ac.uk/enable-your-research/register). Additional genetic data used in this study are not available due to patient privacy and ethical restrictions. The FoundHaplo algorithm and all other supporting data are described in GitHub (https://github.com/bahlolab/FoundHaplo: DOI:10.5281/zenodo.8058286).
